# Optimal dose and pattern of physical activity to prevent diagnosed depression: prospective cohort study

**DOI:** 10.1017/S003329172400343X

**Published:** 2025-02-04

**Authors:** Lars Louis Andersen, Rubén López-Bueno, Aaron Kandola, Rodrigo Núñez-Cortés, Laura López-Bueno, Joaquín Calatayud

**Affiliations:** 1 National Research Centre for the Working Environment, Copenhagen, Denmark; 2 Department of Health Science and Technology, Aalborg University, Aalborg, Denmark; 3 Department of Physical Medicine and Nursing, University of Zaragoza, Zaragoza, Spain; 4 Department of Physiotherapy, Exercise Intervention for Health Research Group (EXINH-RG), University of Valencia, Valencia, Spain; 5 MRC Unit of Lifelong Health and Ageing, University College London, London, UK; 6 Institute of Mental Health, University College London, London, UK; 7 Department of Physical Therapy, Faculty of Medicine, University of Chile, Santiago, Chile

**Keywords:** Accelerometry, Epidemiology, Prevention, Physical activity patterns, UK Biobank, chronic illness, exercise, healthy aging, healthy habits, lifestyle, mental health, public health

## Abstract

**Background:**

Little is known about the dose and pattern of moderate-to-vigorous physical activity (MVPA) to prevent depression. We aimed to assess the prospective association of dose and pattern of accelerometer-derived MVPA with the risk of diagnosed depression.

**Methods:**

We included 74,715 adults aged 40–69 years from the UK Biobank cohort who were free of severe disease at baseline and participated in accelerometer measurements (mean age 55.2 years [SD 7.8]; 58% women). MVPA at baseline was derived through 1-week wrist-worn accelerometry. Diagnosed depression was defined by hospitalization with ICD-10 codes F32.0-F32.A. Restricted cubic splines and Cox regression determined the prospective association of dose and pattern of MVPA with the risk of incident depression.

**Results:**

Over a median 7.9-year follow-up, there were 3,089 (4.1%) incident cases of depression. Higher doses of MVPA were curvilinearly associated with lower depression risk, with the largest minute-per-minute added benefits occurring between 5 (HR 0.99 [95% CI 0.96–0.99]) and 280 (HR 0.67 [95% CI 0.60–0.74]) minutes per week (reference: 0 MVPA minutes).

**Conclusion:**

Regardless of pattern, higher doses of MVPA were associated with lower depression risk in a curvilinear manner, with the greatest incremental benefit per minute occurring during the first 4–5 h per week. Optimal benefits occurred around 15 h/week.

## Introduction

Depression is a prevalent and burdensome mental health disorder, affecting over 300 million people worldwide and contributing significantly to the global burden of disease (Vos et al., 2020; World Health Organization, 2023). Depression significantly impacts healthcare systems through treatment expenses, lost productivity, and long-term care costs (Breslow et al., 2019; Cicek et al., 2022; Johnston et al., 2019; König et al., 2020). The economic burden includes direct medical costs and substantial indirect costs from reduced work performance and absenteeism (König et al., 2020). As a leading cause of disability and diminished quality of life, prevention of depression has long been a public health priority (Cuijpers & Schoevers, 2004). While previous intervention studies suggest that regular physical activity should be an important component in the treatment of depression (Cooney et al., 2013; Schuch et al., 2016), severe depression is difficult to treat and requires substantial resources from healthcare systems. Thus, preventing depression should be a key priority.

Numerous epidemiological studies have explored the association between different doses of physical activity and depression, but findings remain inconsistent. For instance, the Norwegian HUNT study found that up to 2 h of self-reported physical activity per week were associated with reduced risk of depression, whereas larger doses did not appear to confer additional benefits (Harvey et al., 2018). Systematic reviews found that self-reported physical activity from as little as 10 min per day to several hours per week was associated with reduced risk of depression (Mammen & Faulkner, 2013; Pearce et al., 2022; Schuch et al., 2018; Wanjau et al., 2023), leading to uncertainties about the optimal dose. Adding to the uncertainty, the majority of previous studies have relied on self-reported measures of physical activity, which are susceptible to recall bias and measurement error (Dyrstad et al., 2014; Prince et al., 2008).

Besides the total dose of physical activity during the week, information regarding optimal patterns is also relevant, as accumulating physical activity during the weekend may be a more convenient option for some people. Thus, understanding whether these different patterns have similar or different effects on depression risk is important for developing flexible public health recommendations. A cross-sectional analysis from the NHANES study showed similar associations between a self-reported regular activity pattern (daily activity) and a ‘weekend warrior’ activity pattern (accumulating activity on a few days of the week) with depressive symptoms (Liang et al., 2023). However, the cross-sectional nature and reliance on self-reported physical activity limit these findings. A recent study addressed this limitation by using device-measured physical activity data from UK Biobank to examine affective disorders, finding that replacing 30 min of sedentary behavior with moderate physical activity or vigorous physical activity was associated with slightly lower risk of affective disorders (HR’s 0.91–0.95) (Ho et al., 2022). However, their study focused on broader affective disorders rather than specifically diagnosed depression and did not examine different patterns of physical activity accumulation throughout the week. There are several reasons why different activity patterns can be speculated to have either similar or distinct effects on depression risk. Regular, daily activity might provide more consistent mood regulation and stress relief throughout the week, while ‘weekend warrior’ patterns are limited to 1 or 2 days. Conversely, the ‘weekend warrior’ pattern might offer more intense, focused periods of activity that could have different physiological and psychological effects. Weekend activity may also be more likely to involve outdoor exposure or social interaction, potentially influencing its impact on mental health. However, evidence from prospective studies about the importance of different patterns of physical activity and the risk of depression is lacking. To address this issue and the uncertainties from previous studies, large-scale prospective cohort studies with accelerometer-assessed physical activity and robust methods for ascertaining incident depression are needed. Such studies can provide more reliable evidence on whether the pattern of physical activity accumulation throughout the week influences depression risk, independent of total activity volume.

The UK Biobank study, a large-scale prospective cohort study with accelerometer-derived measures of physical activity and comprehensive hospital register follow-up data (Doherty et al., 2017; Littlejohns et al., 2019; Sudlow et al., 2015), provides a unique opportunity to investigate the association of physical activity with the risk of depression. The current study builds upon prior work from the UK Biobank that also used accelerometers to investigate the impact of replacing one type of activity with another on depression (Kandola et al., 2021) and the bi-directional association between physical activity and depression (Choi et al., 2019). In contrast to these previous studies, we employ restricted cubic spline analyses, enabling us to capture potential non-linear associations and identify critical thresholds in the association between moderate-to-vigorous physical activity (MVPA) and depression risk. Furthermore, we analyze whether the pattern of MVPA (regular vs weekend warrior) differentially influences depression risk, providing insights into the importance of activity distribution throughout the week.

This study aims to analyze the optimal dose and pattern of MVPA required to prevent diagnosed depression using objective exposure and outcome in a large sample of adults participating in the UK Biobank study. We hypothesized that there would be a non-linear inverse association between MVPA and risk of diagnosed depression. We also hypothesized that both regular and weekend warrior patterns of MVPA would be associated with reduced depression risk compared to inadequate MVPA.

## Methods

### Study design and population

The UK Biobank study is a large cohort of ~500,000 participants aged 40–69 years enrolled between 2006 and 2010 (Littlejohns et al., 2019). Participants provided informed consent and underwent physical examinations by trained staff and completed touchscreen questionnaires at 22 assessment centers in the UK. The accelerometer sub-study, which forms the basis for our MVPA measurements, took place between June 1, 2013 and December 23, 2015. Follow-up for depression diagnoses continued from the date of accelerometer wear until October 31, 2022 for England, August 31, 2022 for Scotland, and May 31, 2022 for Wales. Ethical approval was obtained from the UK’s National Health Service and National Research Ethics Service (Ethics Committee reference number: 11/NW/0382).

**Figure 1.**
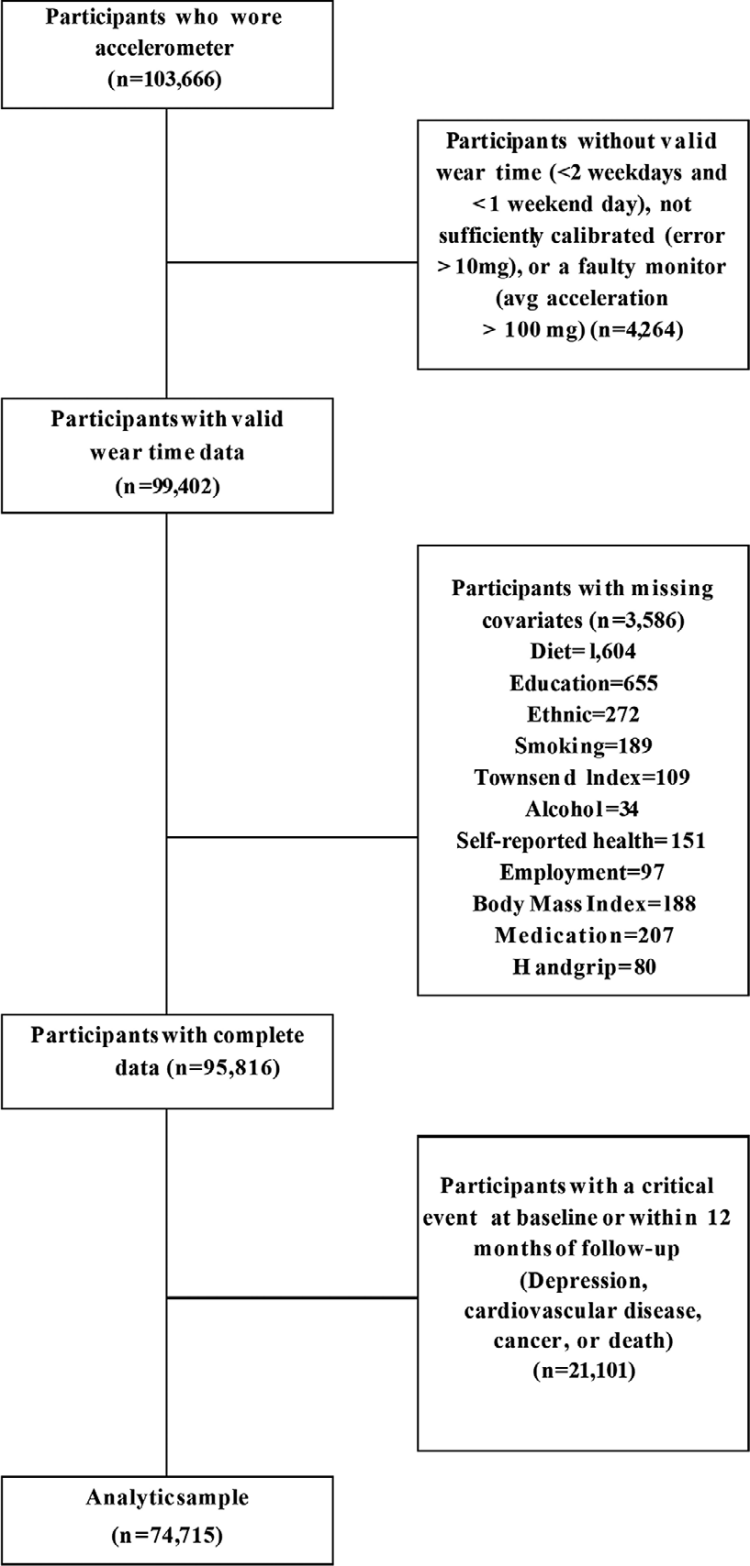
Flowchart of the study sample.

The inclusion criteria for this study were (1) participants aged 40–69 years at UK Biobank enrollment (2006–2010), (2) completion of accelerometer measurements between June 1, 2013 and December 23, 2015, (3) provision of valid accelerometer data (wearing the device for >16 h daily and having at least three valid measurement days, including at least one weekend day), (4) being free of severe disease at baseline (no history of depression, cardiovascular disease, or cancer in medical records) to reduce confounding from conditions that could affect both physical activity levels and depression risk (Celis-Morales et al., 2017; Tian et al., 2023), (5) no diagnosis of depression during the first year after the accelerometer measurement to reduce the risk of reverse causation, and (6) complete data for all covariates included in the analysis. Participants were excluded if they did not meet these criteria or if they had a diagnosis of depression before or at study entry. The flow of participants through the study is shown in Figure 1.

### Exposure: MVPA

The data for this study comprised a cohort of 103,666 participants, constituting the subsample equipped with accelerometers. These individuals utilized Axivity AX3 wrist-worn triaxial accelerometers for 1 week to evaluate MVPA. After calibrating the acceleration signals, the accelerometers captured continuous acceleration data at a sampling rate of 100 Hz, featuring a dynamic range of ±8 g, and segmented the data into 5-s epochs. MVPA was measured as minutes per week spent at 100 milligravities or more, a cut-point supported by previous research (Khurshid et al., 2021; Ramakrishnan et al., 2021).

For the MVPA pattern analysis, we examined a threshold based on current guidelines of aerobic MVPA (≥150 min/week) (Bull et al., 2020). Thereupon, we classified participants as active weekend warrior (WW), when MVPA was equal or above the MVPA threshold and ≥50% of total MVPA was achieved over 1–2 days, active regular when participants achieved MVPA equal or above threshold but not meeting a WW pattern, and inadequately active for participants below MVPA threshold (Kunutsor et al., 2023).

### Outcome: Diagnosed depression

Diagnosed depression was identified as either primary or secondary through the examination of inpatient hospital records retrieved from the UK Biobank. In this study, depression was defined in accordance with the International Classification of Diseases (ICD) 10th edition codes F32.0, F32.1, F32.2, F32.3, F32.4, F32.5, F32.89, F32.9, and F32.A. More information on the outcome is provided in Supplementary eTable 1.

The latest hospital registry-based update was used at the end of follow-up, which corresponded to October 31, 2022, in England; August 31, 2022, in Scotland; and May 31, 2022, in Wales. Participants were censored at these specified dates, upon experiencing the event of interest, or at the time of their death, whichever came first. The death registry included all fatalities occurring before November 30, 2022, in England, Scotland, and Wales.

### Statistical analyses

We examined the dose–response associations of MVPA with depression through restricted cubic splines to allow for potential nonlinearity. To optimally capture the nature and characteristics of the MVPA distribution, we placed knots at the 5th, 50th, and 95th percentiles of MVPA (Desquilbet & Mariotti, 2010). Departure from linearity was checked with a Wald test assessing the null hypothesis that the coefficient of the third spline was equal to zero. According to prior literature (López-Bueno et al., 2023; Marques et al., 2020), the used model was adjusted for the following confounders; age, sex, racial and ethnic background, tobacco use, Townsend Deprivation Index, alcohol consumption, educational attainment, employment status, self-reported health, diet quality, body mass index, medication use (insulin, diabetes, and hypertension), and handgrip strength. Detailed information on these covariates is provided in Supplementary eTable 2. A Wald test showed no interaction between MVPA and any of the aforementioned covariates (*P* > 0.10). We also conducted additional analyses to examine whether there were differences in the association between MVPA and incident depression concerning the MVPA pattern (i.e. weekend-warrior vs active regular) through a fully adjusted Cox regression and estimates presented in a forest plot. The number of missing data was quite low in subsample with accelerometer measurements (Figure 1), and we therefore used complete cases for all analyses. Estimations are shown with 95% confidence intervals (CIs). Analyses were conducted from April to May 2024 using Stata version 18.0 (StataCorp).

## Results

The final study sample comprised 74,715 individuals (mean [SD] age, 55.2 [7.8] years; 58% of whom were women) who wore accelerometers for 1 week, spanning from any timepoint between June 1, 2013 and December 23, 2015. Table 1 displays information on the characteristics of the study sample.

**Table 1.** Sample characteristics at baseline by physical activity pattern

	Inadequately active^a^	Weekend warrior^a^	Active regular^a^	Total
*N*	23,994 (32.1%)	29,994 (40.1%)	20,727 (27.7%)	74,715 (100.0%)
Age, mean (SD), y	55.9 (7.9)	55.2 (7.7)	54.2 (7.8)	55.2 (7.8)
Sex				
Male	7,582 (31.6%)	14,250 (47.5%)	9,791 (47.2%)	31,623 (42.3%)
Female	16,412 (68.4%)	15,744 (52.5%)	10,936 (52.8%)	43,092 (57.7%)
Townsend deprivation index^b^	−1.8 (2.8)	−1.9 (2.7)	−1.3 (4.0)	−1.7(2.8)
Ethnic background^c^				
White	23,072 (96.2%)	29,186 (97.3%)	20,007 (96.5%)	72,265 (96.7%)
Black	96 (0.4%)	74 (0.2%)	74 (0.4%)	244 (0.3%)
Asian	376 (1.6%)	293 (1.0%)	250 (1.2%)	919 (1.2%)
Other	450 (1.9%)	441 (1.5%)	396 (1.9%)	1,287 (1.7%)
Tobacco use				
Never	13,675 (57.0%)	18,143 (60.5%)	12,107 (58.4%)	43,925 (58.8%)
Former	8,305 (34.6%)	10,192 (34.0%)	7,309 (35.3%)	25,806 (34.5%)
Current	2,014 (8.4%)	1,659 (5.5%)	1,311 (6.3%)	4,984 (6.7%)
Alcohol consumption				
Never	962 (4.0%)	670 (2.2%)	525 (2.5%)	2,157 (2.9%)
Former	762 (3.2%)	684 (2.3%)	562 (2.7%)	2,008 (2.7%)
Current	22,270 (92.8%)	28,640 (95.5%)	19,640 (94.8%)	70,550 (94.4%)
Self-reported health				
Excellent	3,813 (15.9%)	7,889 (26.3%)	5,561 (26.8%)	17,263 (23.1%)
Good	14,496 (60.4%)	18,337 (61.1%)	12,334 (59.5%)	45,167 (60.5%)
Fair	4,755 (19.8%)	3,409 (11.4%)	2,562 (12.4%)	10,726 (14.4%)
Poor	930 (3.9%)	359 (1.2%)	270 (1.3%)	1,559 (2.1%)
Employed				
Yes	14,492 (60.4%)	19,408 (64.7%)	14,210 (68.6%)	48,110 (64.4%)
No	9,502 (39.6%)	10,586 (35.3%)	6,517 (31.4%)	26,605 (35.6%)
Body mass index, mean (SD), kg/m^2^	27.912 (5.154)	26.058 (3.930)	25.817 (4.052)	26.587 (4.486)
Family history of depression				
No	21,684 (90.4%)	27,188 (90.6%)	18,592 (89.7%)	67,464 (90.3%)
Yes	2,310 (9.6%)	2,806 (9.4%)	2,135 (10.3%)	7,251 (9.7%)
Educational attainment, mean (SD), y	13.7 (5.3)	15.0 (5.3)	15.2 (5.3)	14.6 (5.3)
Diet quality				
Good	5,006 (20.9%)	6,898 (23.0%)	5,197 (25.1%)	17,101 (22.9%)
Intermediate	12,927 (53.9%)	16,230 (54.1%)	10,912 (52.6%)	40,069 (53.6%)
Poor	6,061 (25.3%)	6,866 (22.9%)	4,618 (22.3%)	17,545 (23.5%)
Insulin medication				
No	20,552 (85.7%)	26,949 (89.8%)	18,759 (90.5%)	66,260 (88.7%)
Yes	3,442 (14.3%)	3,045 (10.2%)	1,968 (9.5%)	8,455 (11.3%)
Blood pressure medication				
No	23,784 (99.1%)	29,873 (99.6%)	20,625 (99.5%)	74,282 (99.4%)
Yes	210 (0.9%)	121 (0.4%)	102 (0.5%)	433 (0.6%)
Cholesterol medication				
No	19,569 (81.6%)	26,238 (87.5%)	18,383 (88.7%)	64,190 (85.9%)
Yes	4,425 (18.4%)	3,756 (12.5%)	2,344 (11.3%)	10,525 (14.1%)
Handgrip, mean (SD), kg	30.3 (10.8)	33.8 (11.2)	33.8 (10.9)	32.7 (11.1)
MVPA, mean (SD), weekly minutes	73.8 (45.7)	353.1 (189.7)	466.1 (265.0)	294.7 (244.5)
LPA, mean (SD), weekly minutes	2059.4 (796.6)	2160.4 (671.8)	2152.8 (699.8)	2125.3 (723.6)
Sedentary, mean (SD), weekly minutes	4042.2 (947.1)	3890.0 (722.6)	3790 (780.5)	3913.8 (823.8)

a Inadequately active defined as MVPA below the recommended threshold of 150 minutes per week. Weekend warrior defined as MVPA equal or above the MVPA threshold and ≥50% of total MVPA achieved over 1–2 days. Active regular defined as MVPA equal or above threshold but not meeting a weekend warrior pattern.

b Represents self-reported “ethnic background.” Race classification of “Other” defined as self-report of a race other than Asian, Black, or White.

c The Townsend Deprivation Index is a way to measure material deprivation standardized by geographic area. Greater values indicate more deprivation. The sample range is −6.3 to 10.6, with values around −2 and −1 indicating somewhat less deprivation compared to average based on geographic location.

LPA, Light physical activity; MVPA, Moderate-to-vigorous physical activity.

The median follow-up time was 7.9 years (interquartile range [IQR] 7.3–8.4). During the follow-up period, there were 3089 (4.1%) incident cases of diagnosed depression.

**Figure 2.**
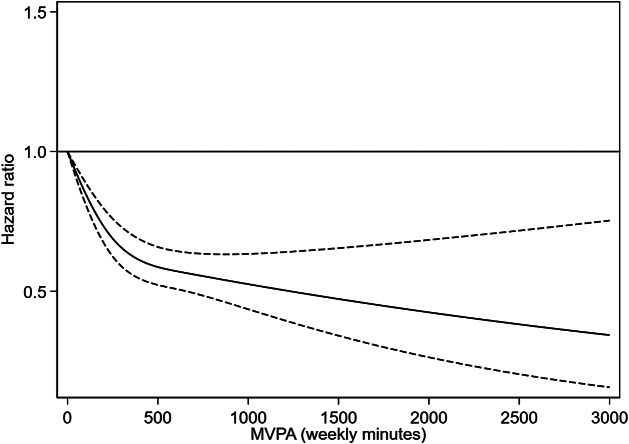
Dose–response associations between moderate to vigorous physical activity (MVPA) and diagnosed depression. Model adjusted for age, sex, racial and ethnic background, tobacco use, Townsend Deprivation Index, alcohol consumption, educational attainment, employment status, self-reported health, diet quality, body mass index, medication use, and handgrip. Reference: 0 weekly minutes of MVPA. Dotted lines depict 95% CIs.

Figure 2 depicts the dose–response association between MVPA and diagnosed depression. Higher doses of MVPA were curvilinearly associated with reduced depression risk, with the steepest minute-per-minute added benefits occurring between 5 (HR 0.99 [95% CI 0.96–0.99]) and 280 (HR 0.67 [95% CI 0.60–0.74]) minutes per week (reference: 0 MVPA minutes). The lowest value of the upper 95% CI was observed at 875 min per week (HR 0.54 [95% CI 0.46–0.63]), which we therefore defined as the optimal dose. Beyond this point, HRs continued to decrease until 3000 min per week (HR 0.34 [95% CI 0.16–0.75]), but the 95% CI widened to values higher than that observed at 875 min per week. In addition, to provide more realistic values for the general population, we conducted an sensitivity analysis of the dose-response association restricted to 1500 min/week of MVPA, which yielded similar trajectories and is presented in Supplementary eFigure 1.

**Figure 3.**
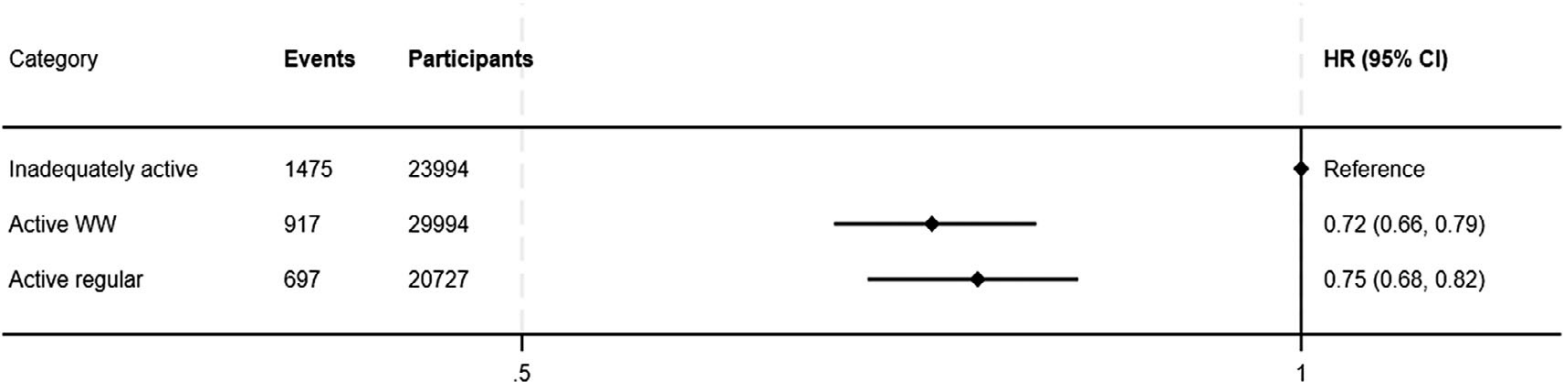
Associations of pattern of moderate to vigorous physical activity (MVPA) with incident depression. Three physical activity patterns are compared: inadequately active (reference), active weekend warrior (WW) (MVPA equal or above 150 weekly minutes and ≥50% of total MVPA achieved over 1-2 days), and active regular (achieving MVPA equal or above 150 weekly minutes without following a WW pattern). Bars depict 95% CIs. Model adjusted for age, sex, racial and ethnic background, tobacco use, Townsend Deprivation Index, alcohol consumption, educational attainment, employment status, self-reported health, diet quality, body mass index, medication use, and handgrip. CI, confidence interval; HR, hazard ratio

Figure 3 shows that weekend warrior (HR 0.72 [95% CI 0.66–0.79]) and active regular (HR 0.75 [95% CI 0.68–0.82]) patterns were quite similarly associated with reduced risk of incident depression (reference: inadequately active).

## Discussion

This study investigated the dose–response relationship and patterns of accelerometer-assessed MVPA associated with the risk of incident depression in a large cohort of UK adults. Our findings reveal a particularly steep reduction in depression risk when individuals transition from no MVPA to even modest amounts of MVPA. This highlights the critical public health message that any increase in MVPA, even at low doses, can yield significant mental health benefits. Our findings also suggest that higher volumes of MVPA are associated with a reduced risk of diagnosed depression in a curvilinear manner, with the greatest minute-per-minute benefits occurring during the first 4–5 h per week and optimal benefits observed at around 15 h per week. Furthermore, we found that both weekend warrior and regular MVPA patterns provide similar protective effects against depression compared to being inadequately active.

Our results expand on previous systematic reviews that have found self-reported physical activity to be associated with a reduced risk of depression (Mammen & Faulkner, 2013; Pearce et al., 2022; Schuch et al., 2018; Wanjau et al., 2023). Our study extends these findings by providing a more precise estimate of the dose-response relationship using accelerometer-assessed MVPA. The curvilinear association observed in our study suggests that the protective effects of MVPA against depression may have a ceiling effect at around 15 h per week, although the wide confidence intervals beyond this point create uncertainty as to whether even larger doses may possibly confer even greater benefits. However, from a practical point of view, public health recommendations of such large doses of MVPA are probably not feasible. Interestingly, the Norwegian HUNT study found that up to 2 h of self-reported physical activity per week was associated with a reduced risk of depression, while larger doses did not appear to confer additional benefits (Harvey et al., 2018). The marked difference in findings between these studies highlights the importance of using objective measures of exposure and outcomes to reach valid conclusions. Our findings regarding the protective association between MVPA and mental health outcomes align with those reported by Ho and coworkers, who found similar protective associations between device-measured physical activity and broader affective disorders in the UK Biobank cohort (Ho et al., 2022). However, our study extends these findings by demonstrating that these protective associations specifically apply to diagnosed depression and persist regardless of whether MVPA is accumulated in a regular pattern throughout the week or concentrated on weekends.

Based on a comprehensive review by Kandola and colleagues, physical activity exerts antidepressant effects through multiple biological, psychological, and social mechanisms (Kandola et al., 2019). Key biological pathways include promoting neuroplasticity, reducing inflammation and oxidative stress, and regulating the neuroendocrine system. Physical activity also provides psychosocial benefits such as improved self-esteem, social support, and self-efficacy that may help alleviate depressive symptoms. The authors note that while the understanding of these mechanisms is growing, more research directly investigating them in people with depression is needed to establish their relative importance and identify factors that moderate the antidepressant effects of exercise on an individual basis.

The similar protective benefits observed for weekend warrior and regular MVPA patterns in our study are consistent with a previous cross-sectional analysis from the NHANES study, which found comparable associations of self-reported regular and weekend warrior activity patterns with depressive symptoms (Liang et al., 2023). In line with this, a previous cohort study with data from the US National Health Interview Survey reported no differences between these two physical activity patterns to reduce all-cause and cause-specific mortality rates (dos et al., 2022). A meta-analysis based on questionnaire-assessed physical activity found that leisure-time and transport-related physical activities were associated with reduced risk of mental health problems, while the opposite was the case for work-related physical activity (White et al., 2017). Weekend MVPA is more likely to be leisure-based, while weekday activity often includes occupational, domestic, and transportation-related physical activity. The similar protective effects observed for weekend warriors and regular exercisers in the present study suggest that the total volume of MVPA is more important than its distribution throughout the week. However, our accelerometer data does not distinguish between different domains of MVPA. Future research using both accelerometry and detailed activity logs could help disentangle the effects of different physical activity domains and patterns on depression risk. This could provide valuable insights for tailoring physical activity recommendations to maximize mental health benefits. Nevertheless, our findings have important public health implications, as they suggest that individuals who struggle to maintain a regular physical activity routine due to time constraints or other barriers may still benefit from accumulating MVPA on a few days of the week. Promoting MVPA as a flexible and achievable goal, rather than a rigid prescription, may help to increase adherence and reduce the burden of depression at a population level.

We also compared sedentary time and light physical activity (LPA) between the groups. This showed minimal differences between weekend warriors and those with regular activity patterns, suggesting that the protective effects of MVPA against depression are not substantially influenced by differences in sedentary behavior between these groups. In addition, we performed sensitivity analyses controlling for sedentary time and LPA, which did not change the main conclusion (results not shown). This finding strengthens that the total volume of MVPA, rather than its distribution throughout the week or associated differences in other movement behaviors, may be the key factor in reducing depression risk.

The strengths of our study include the large sample size, the use of highly reliable data sources, including accelerometer-assessed MVPA, and the robust ascertainment of incident depression through hospital registers. Nevertheless, limitations also exist. First, individuals might have modified their behavior during the MVPA measurement period. Second, the UK Biobank is not representative of the general UK population due to volunteer bias, lack of ethnic diversity, higher socioeconomic status, healthy volunteer effect, and potential geographic coverage issues. Third, information on the covariates was collected several years prior to the use of accelerometry in many cases, which along with the fact that we have analyzed pooled diagnosed depression (i.e. different depression grades together), may pose a risk of misclassification bias for the respective covariates and outcomes. Fourth, MVPA measured through accelerometry does not account for stationary exercise or light activity, which should be considered to better interpret the findings of the present study (Kandola et al., 2020). Fifth, even though the study has accounted for a large set of potential confounders, there is still a risk for residual confounding bias concerning psychological factors that may affect the severity of depression symptoms. Sixth, while MVPA guidelines and population behaviors may have evolved since the initial data collection, we used current guidelines to ensure our findings are applicable to present-day public health recommendations. The objective measurement of MVPA via accelerometry provides a consistent metric across time. Seventh, our analysis focused on MVPA without adjusting for light physical activity or sedentary behavior. Future research using compositional data analysis approaches could provide insights into the relative importance of different movement behaviors for depression risk. Eight, while excluding participants with CVD and cancer helped isolate the association between MVPA and depression, it may limit the generalizability of our findings to populations with these comorbidities. Finally, reverse causation may have influenced our results, as individuals with subclinical depression may have been less likely to engage in MVPA at baseline. However, we minimized this bias by excluding participants with a history of depression or other severe chronic conditions at baseline.

Higher doses of MVPA were associated with lower depression risk in a curvilinear manner, with the greatest minute-per-minute incremental benefits occurring during the first 4–5 h per week. Optimal benefits occurred around 15 h/week. Both weekend warrior and regular MVPA patterns provided similar protective effects against depression compared to being inadequately active, providing greater freedom of choice in weekly MVPA planning. These findings highlight the importance of promoting MVPA as a key strategy for preventing depression and suggest that flexible approaches to increasing MVPA levels may be effective in reducing the burden of this prevalent and debilitating mental health disorder. The present findings have the potential to inform public health guidelines and interventions aimed at promoting MVPA as a preventive strategy for depression.

## Supporting information

Andersen et al. supplementary material Andersen et al. supplementary material

## Supplementary material

The supplementary material for this article can be found at http://doi.org/10.1017/S003329172400343X.
