# Supplementary material for: Optimal dose and pattern of physical activity to prevent diagnosed depression: prospective cohort study
Source: Psychol Med. 2025 Feb 4;55:e2. doi: 10.1017/S003329172400343X (PMC11968116; doi:10.1017/S003329172400343X)
Supplement: Andersen et al. supplementary material [file S003329172400343Xsup001.docx]

**Supplemental Online Content**

**eTable 1.** Diagnosed depression definitions

**eTable 2.** Covariate definitions

**eFigure 1.** Dose‒response associations between moderate to vigorous physical activity (MVPA) and diagnosed depression restricted to individuals doing 1500 min/week of MVPA or lower

**eReferences**

| **eTable 1. Diagnosed definitions** | | | | |
| --- | --- | --- | --- | --- |
| **Phenotype** | **Data fields** | **Field names** | **Data codes** | **Data code definitions** |
| Depression | 41202  41204 | Diagnoses - main ICD10  Diagnoses – secondary ICD10 | Diagnosis, main or secondary:  -ICD-10:  F32.0, F32.1, F32.2, F32.3, F32.4, F32.5, F32.89, F32.9, F32.A | Major depressive disorder, single episode, mild  Major depressive disorder, single episode, moderate  Major depressive disorder, single episode, severe without psychotic features  Major depressive disorder, single episode, severe with psychotic features  Major depressive disorder, single episode, in partial remission  Major depressive disorder, single episode, in full remission  Other specified depressive episodes  Major depressive disorder, single episode, unspecified  Depression, unspecified |
| ICD = International Statistical Classification of Diseases and Related Health Problems | | | | |

| **eTable 2. Covariate definitions** | | | | |  |
| --- | --- | --- | --- | --- | --- |
| **Covariate** | **UK**  **Biobank Field ID(s)** | **UK Biobank Field Name(s)** | **Instance(s)** | **Coding** | **Notes** |
| Age | 21022 | Age | 0 | Continuous age in years | Age at recruitment |
| Sex | 31 | Sex | 0 | 1)Male  2)Female |  |
| Ethnic background* | 21000 | Ethnic background | 0, 1† | 1)White  2)Black  3)Asian  4)Other | Response 5 (Chinese) included under category Asian. Responses 2 (Mixed) and 6 (Other ethnic group) combined into category Other given low frequency |
| Tobacco use* | 20116 | Smoking status | 0, 1† | 1) Current  2) Previous  3) Never |  |
| Townsend deprivation index | 22189 | Townsend deprivation index at recruitment | 0 | Continuous scale |  |
| Alcohol use* | 20117 | Alcohol drinker status | 0, 1† | 1 ) Current  2) Previous  3) Never |  |
| Diet quality* | 1289  1299  1309  1319  1369  1379  1389  1349  1478 |  | 0, 1† | 1 ) Healthy  2) Intermediate  3) Unhealthy | Diet quality categorized as follows (see Bhattacharya et al.^1^):  - Unhealthy: a) below-median intake of fruits and vegetables and b) above- median intake of red meat or processed meat or above-median frequency of adding salt to their diet  - Healthy: a) above-median intake or fruits and vegetables and b) below- median intake of red meat or processed meat and below-median frequency of adding salt to their diet  - Intermediate: not classified as healthy or unhealthy |
| Educational attainment* | 6138 | Qualifications | 0, 1† | Continuous educational attainment in years | Degree/qualification status and reported age completed full time education were converted to years of educational attainment as follows (see Okbay et al.^2^):  - College or university degree: 20  - Advanced Subsidiary/Advanced Levels or equivalent: 13  - Ordinary Levels/General Certificate of Secondary Education or equivalent: 10  - Certificate of Secondary Education or equivalent: 10  - National Vocational Qualification or Higher National Diploma or Higher National Certificate or equivalent: Age completed full time education – 5  - Other professional qualification: 15  - None of the above: 7 |
| Self-reported health* | 2178 | Overall health rating | 0, 1† | 1) Excellent  2) Good  3) Fair  4) Poor |  |
| Employment status* | 6142 | Current employment status | 0, 1† | 1) Paid employment/Self- employed  2) Retired or  unemployed | *In paid employment or self-employed* coded as Paid employment/Self-employed, all other answers coded as Retired or unemployed |
| Body mass index | 21001 | Body mass index | 0, 1† | Continuous in kg/m^2^ |  |
| Medication use* | 6153  6177 | Medication for cholesterol, blood pressure, or diabetes | 0, 1† | 0) No  1) Yes |  |
| All-cause cancer | 41202  41204  40006  40001  40002 | Diagnoses - main ICD10  Diagnoses – secondary ICD10  Type of cancer: ICD10  Underlying (primary) cause of death: ICD10  Contributory (secondary)  cause of death: ICD10 | 0 | ICD10: C0, C1, C2, C3, C4 (excluding C49.9), C5, C6, C70, C71, C72, C73, C74, C75, C7A, C8, C9 | Coded as yes or no |
| Cardiovascular disease | 41202  41204  40001  40002  41282 | Diagnoses - main ICD10  Diagnoses – secondary ICD10  Underlying (primary) cause of death: ICD10  Contributory (secondary)  cause of death: ICD10  Operative procedures – main OPCS  Operative procedures – secondary OPCS | 0 | 0) No  1) Yes | Following the study by Tikkanen et al,^3^ positive response to cardiovascular disease was coded as Yes if participants matched with any of the following codes in Diagnosis, main or secondary, or Cause of death, primary or secondary:  -ICD-10: I20.0, I21, I22, I63, I60, I62, I48  -ICD-9: 410, 411, 433, 434, 430, 431, 432, 428, 450, 427.3  Positive response to any cardiovascular disease was coded as Yes if participants matched with any of the following codes in Operative procedures, main or secondary:  -K40, K41, K42, K43, K44, K45, K46, K49, K50, K50.1, K62.2, K62.3, K62.4, K75 |
| Handgrip | 46  47 | Handgrip strength (right)  Handgrip strength (left) | 0, 1† | Continuous in kg | Maximum value of either hand |
| *Responses *Do not know* and *Prefer not to answer* considered non-informative (i.e., missing)  †Instance 0 = baseline assessment (3/2006-10/2010), Instance 1 = first follow-up assessment (12/2009-6/2013). Priority was given to Instance 1 data when available given closer temporal proximity before accelerometry  ICD = International Statistical Classification of Diseases and Related Health Problems | | | | | |

**eFigure 1. Dose‒response associations between moderate to vigorous physical activity (MVPA) and diagnosed depression restricted to individuals doing 1500 min/week of MVPA or lower**

**
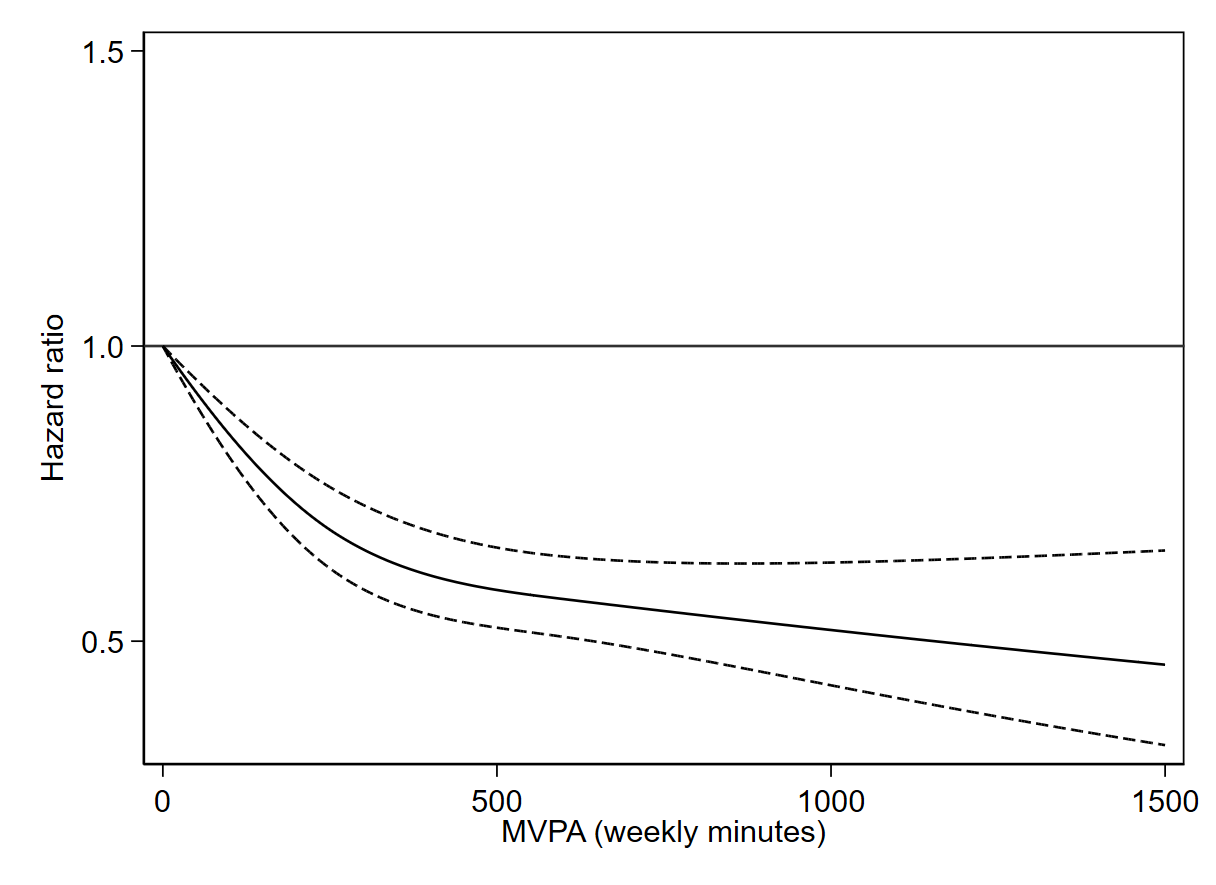
**

Model adjusted for age, sex, racial and ethnic background, tobacco use, Townsend Deprivation Index, alcohol consumption, educational attainment, employment status, self-reported health, diet quality, body mass index, medication use, and handgrip. Reference: 0 weekly minutes of MVPA. Dotted lines depict 95%CIs.

**eReferences**

1. Bhattacharya R, Zekavat SM, Uddin MM, et al. Association of Diet Quality With Prevalence of Clonal Hematopoiesis and Adverse Cardiovascular Events. JAMA Cardiol. 2021;6(9):1069-1077.

2. Okbay A, Wu Y, Wang N, et al. Polygenic prediction of educational attainment within and between families from genome-wide association analyses in 3 million individuals. Nat Genet. 2022;54(4):437-449.

3. Tikkanen E, Gustafsson S, Ingelsson E. Associations of fitness, physical activity, strength, and genetic risk with cardiovascular disease: Longitudinal analyses in the UK biobank study. Circulation. 2018;137(24):2583–91.
